# Posterior ankle arthroscopic treatment of a talar chondroblastoma with allograft and a platelet-rich plasma-fibrin glue: A case report and literature review

**DOI:** 10.3389/fsurg.2022.1039785

**Published:** 2023-01-05

**Authors:** Cheng Chen, ZhenDong Li, JianFeng Xue, ZhongMin Shi

**Affiliations:** Foot & Ankle Section, Department of Orthopaedics, Shanghai Jiao Tong University Affiliated Sixth People’s Hospital, Shanghai, China

**Keywords:** arthroscopy, chondroblastoma, talus, benign bone tumor, platelet-rich plasma-fibrin glue, allograft

## Abstract

Talar chondroblastoma, which is a benign tumor of immature cartilage cells, is an uncommon but easily missed diagnosis of foot pain. Arthroscopic treatment for this condition is a safe, powerful, and promising technique with definitive advantages of visualization and minimal invasion. Here, we report a case of a talar chondroblastoma treated by posterior ankle arthroscopic curettage, allograft bone graft, and platelet-rich plasma-fibrin glue (PRP-FG) application.

**Level of evidence:**

Case Report. Level IV.

## Introduction

Chondroblastoma accounts for 1% of all bone tumors (1), which primarily affect a patient population aged 10–30 years old. Chondroblastoma is generally classified as a benign but “intermediate, rarely metastasizing” category of tumor with a recurrence rate of 4.8%–39.5% (2–4). The first case of chondroblastoma was reported in the year 1928 and the first talar chondroblastoma was reported in 1947 (5, 6). Typically, chondroblastoma occurs in the epiphysis of a long bone, but less than 10% affects the talus (7, 8). The talar chondroblastoma reported in literature were mainly located at the body of the talus, which uncommonly causes cortical erosion. Surgery is the main treatment modality for chondroblastoma, including curettage, resection, and radiofrequency ablation alone or in combination with other procedures (2, 9). Surgery for talar chondroblastoma is technically challenging due to the risk of joint surfaces breaking or fracture of the talus. Given the small size of the talus with the deep and complicated location of the tumor, it is very difficult to expose the operative field and excise it if open surgery is performed. Compared with open surgery, arthroscopic treatment has obvious advantages of visualization and minimal invasion, allowing for early mobilization and superior postsurgical functional and cosmetic results.

To the best of our knowledge, only one case of a patient who underwent posterior ankle arthroscopic treatment for a talar chondroblastoma has been reported (10). Here, we report a talar chondroblastoma patient treated by posterior ankle arthroscopic curettage, allograft bone graft, and platelet-rich plasma-fibrin glue (PRP-FG) application.

## Case presentation

A 32-year-old man presented with pain and swelling in his right ankle on bearing weight and walking for 7 months. The only remarkable clinical finding was diffuse tenderness over the posterior ankle and deep-seated pain at maximal plantar flexion, which could not be exactly pinpointed. The Visual Analogue Scale (VAS) score was 6 points and the American Orthopedic Foot and Ankle Society (AOFAS) score was 71 points.

**Figure 1:**
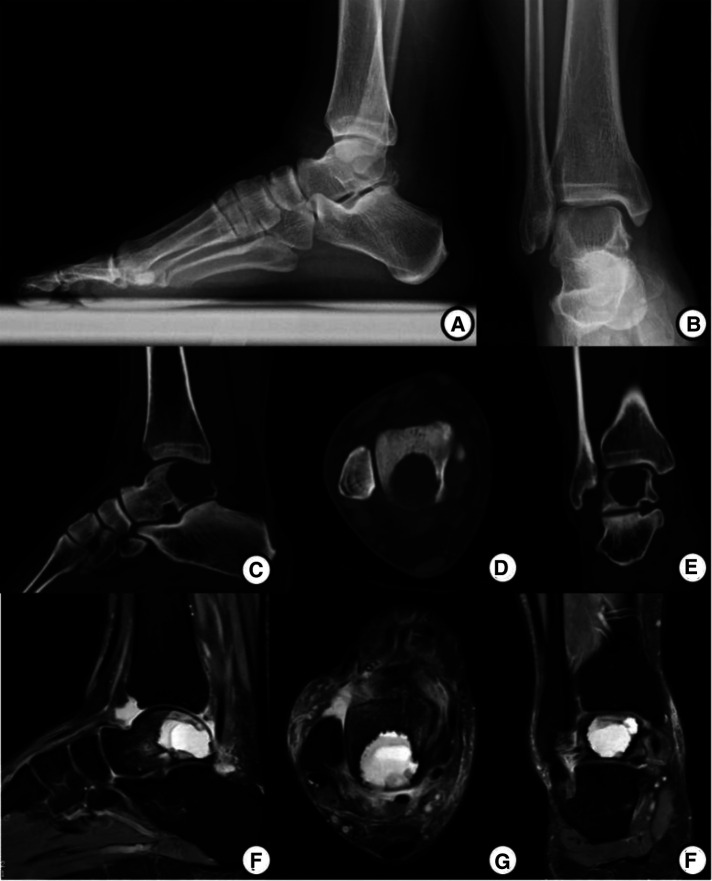
Preoperative imagological data. (**A,B**) AP and Lateral x-ray before operation show a well-demarcated and lytic lesion in the posterior talus. (**C**–**E**) Preoperative CT images show a lesion. (**F**–**H**) Preoperative MRI images show a lesion with no cortical and articular surface breakthrough, measuring 2.5*2.2*2.2 cm.

**Figure 2:**
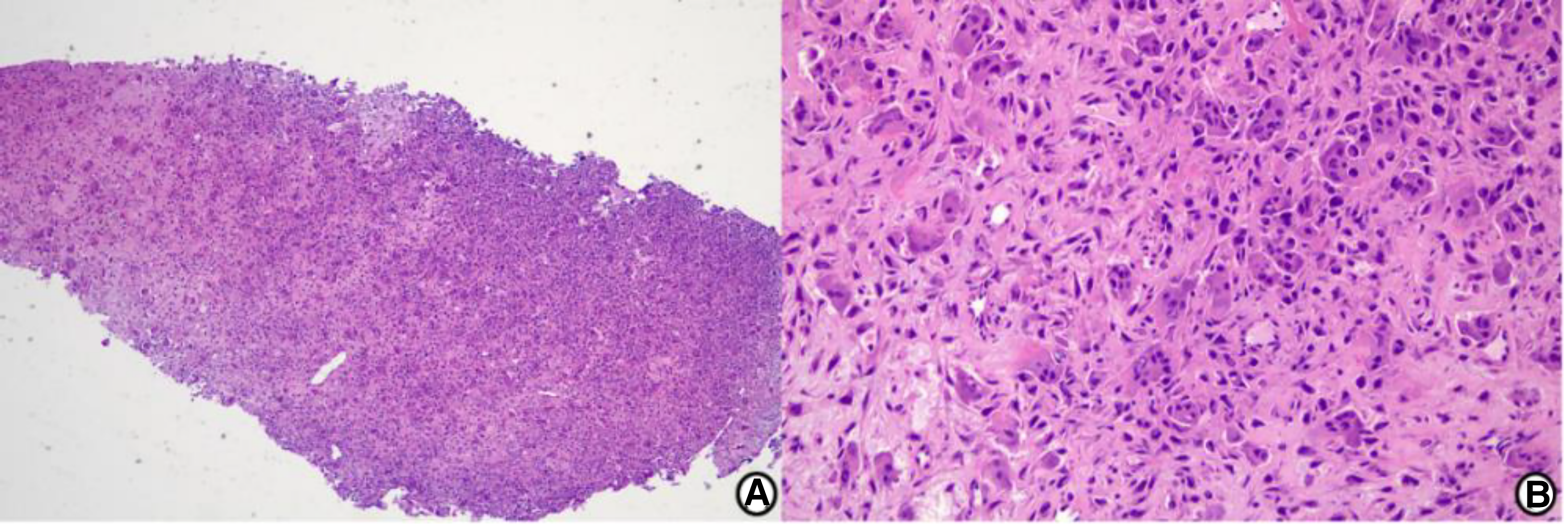
Histological image. Histological examination shows chondroblastoma cells and foci of the eosinophilic chondroid matrix. The immunohistochemical results show KI67(5% +), H3F3A(−), Kp1(+), CD163(partial +), H3F3B(+), P16(partial +), P63(partial +), and SATB2(+). The findings are consistent with chondroblastoma. (**A**) Low-power view (hematoxylin and eosin stain, origin magnification ×40). (**B**) High-power view (hematoxylin and eosin stain, origin magnification ×200).

Preoperative x-ray and CT images showed a well-demarcated lytic lesion in the posterior half of the talar body. Preoperative MRI images showed a lesion with no cortical and articular surface breakage, measuring 2.5*2.2*2.2 cm (Figure 1). An x-ray-guided punch biopsy was performed and both histopathology and immunohistochemical results confirmed the diagnosis of chondroblastoma (Figure 2).

**Figure 3:**
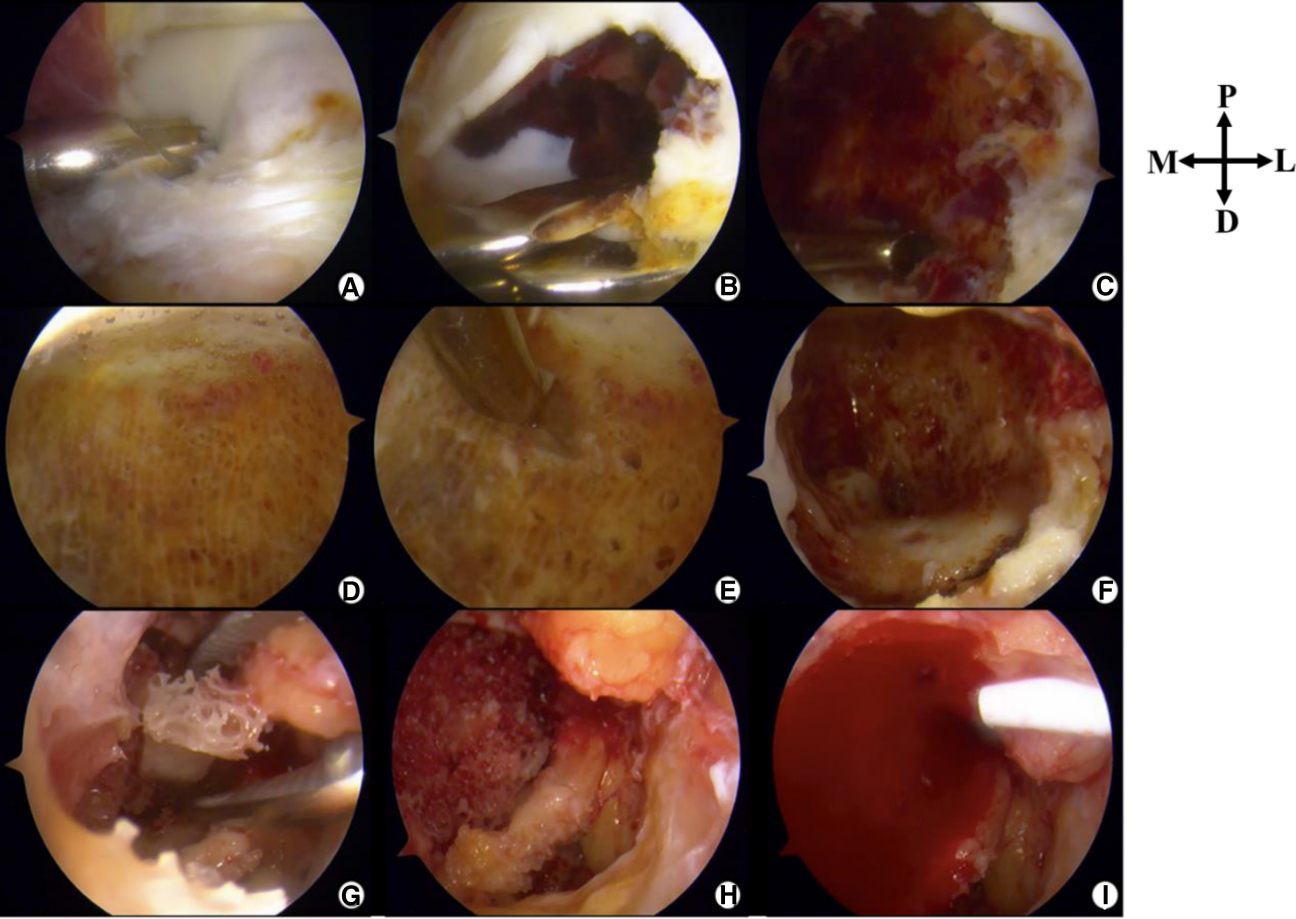
Intraoperative photographs (posterior ankle arthroscopy). P, Proximal; D, Distal; M, Medial; L, Lateral. (**A**) The lesion is located anterior to the posterior intermalleolar ligament. (**B**) Parts of the cartilage are removed to disclose the lesion. (**C**) Surgical debridement of the lesion followed by curettage. (**D**) Healthy cancellous bone after curettage. (**E**) Microfracture. (**F**) “Dry” arthroscopic view showing the cavity. (**G**) Allograft bone graft. (**H**) A photograph after the completion of implantation of allograft bone. (**I**) PRP-FG (platelet rich-plasma-fibrin glue) was injected.

After careful evaluation and discussion with the patient, we finally chose the posterior ankle arthroscopic treatment option. Arthroscopy was conducted 2 weeks after the biopsy procedure. The conditions are described in Figure 3.

After spinal anesthesia, the patient was placed in a prone position with a thigh tourniquet. All bony prominences were protected with cushion jelly to prevent pressure sore. A total of 40 ml blood was collected from his arm vessel. PRP was prepared using the package produced by the Shandong Weigao Group Medical Polymer Company. A quantity of 4 ml of concentrated PRP was prepared by the double centrifugation method at 2,000 r/min for 10 min with a centrifugation radius of 15 cm. Concentrated PRP was set aside.

The 2-portal posterior ankle arthroscopy technique, first introduced by Van Dijk (11), was used with a saline gravity irrigation system. A 4.0-mm, 30° arthroscope was inserted via the posterolateral portal and a shaver was placed in the posteromedial portal. By identifying the flexor hallucis longus by protecting its medial aspect, the neurovascular bundle was kept safe. After debridement of the posterior ankle capsule and posterior intermalleolar ligament, the surface of the lesion with overlying soft cartilage was identified. The soft cartilage at the most posterior part of the talar dome (avoiding the weight-bearing area) was removed to expose the lesion, which was soft and friable and grey in color, mixed with a dark red–stained hematoma. It was curetted until the healthy cancellous bone was exposed. Microfracture was conducted on the wall of the cavity. After thorough lavage, the saline inflow was stopped and the intra-articular fluid was drained out. Then, we switched to “dry” transosseous arthroscopy. Under direct vision, 30 g allograft granular cancellous bone was packed into the lesion and gently tamped down. Once the bone graft was done, concentrated PRP fibrinogen and thrombin (Zhejiang Hangkang Pharmaceutical Co., Ltd.) were mixed. Then, the injection was applied to the surface defect and fibrin glue (Harbin Pacific Biopharmaceutical Co., Ltd.) was used for sealing. The portals were closed with a suture, and a dry, sterile compression dressing was applied. The foot and ankle were immobilized with a splint.

The suture was removed 2 weeks after the surgery. From the first day to postoperative 2 weeks, the patient remained on non-weight-bearing crutches with the ankle was placed in 90° dorsiflexion. Active muscle contraction was encouraged, and the range of motion was restricted within the first 2 weeks. Ankle plantar flexion and dorsiflexion exercises were started after 2 weeks, with the number increased by 30 times a day until it reached 150 times a day. Then, the partial weight-bearing process with a postoperative walking boot and crutch was gradually increased from 2 to 6 weeks. This process started from a weight of 20 lbs, with the weekly weight increased by 20 lbs. At the postoperative 6 weeks (the first follow-up visit), an x-ray revealed an initially incorporated bone graft. We encouraged the patient to increase his weight-bearing walking exercise and activities as tolerated. Instructions on rehabilitation exercises were given to the patient during follow-up visits.

**Figure 4:**
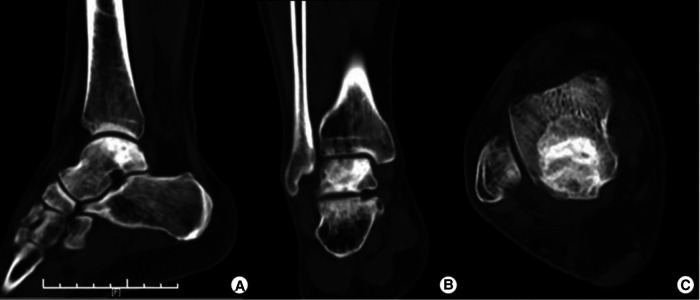
One-year postoperative CT images showing good bone remodeling and osteointegration with no tumor recurrence.

We found that there were no postoperative complications. The first follow-up was conducted at 6 weeks. Since then, follow-ups have been conducted every 3 months. The most recent follow-up was performed one year after the surgery. The patient was satisfied with their full motor function recovery and pain-free condition. The VAS score was 0 points, and the AOFAS score was 100 points. Ankle flexion and extension range of motion was 70°. One-year postoperative CT images showed good bone remodeling and osteointegration with no tumor recurrence (Figure 4).

## Discussion

To the best of our knowledge, only one case of a patient with posterior ankle arthroscopic treatment for a talar chondroblastoma has been reported. Yonter et al. (10) reported a patient with a 1.3*2.0*0.8 = 2.08 cm^3^ chondroblastoma in the posterior talus via arthroscopic curettage, allograft bone graft, and augmentation with a cell-free matrix. Our case presented a challenging larger-sized talar chondroblastoma (2.5*2.2*2.2 = 12.1 cm^3^), and we combined posterior ankle arthroscopy, including wet and “dry” transosseous arthroscopy, with curettage, allograft bone graft, and PRP-FG. Our patient showed satisfactory results without any recurrence at a 1-year follow-up.

Although talar chondroblastoma is uncommon, almost 50% of foot chondroblastoma occurs in the talus (12). Interestingly, Angelini et al. (12) suggested that the recurrence rate of chondroblastoma in the foot was even lower than that in other body locations. There are few reports about appropriate treatments and clear outcomes for talar chondroblastoma. Therefore, we review and summarized the literature in Table 1. We found that most of the cases reported a generally good prognosis, except the case of a patient who had a massive tumor with local invasion (talus, calcaneus, tibiotalar joint, and the adjacent soft tissue).

**Table 1:** Treatment and outcome of talar chondroblastoma previously reported in the literature.

Ref	Patient	Side	Lesion	Treatment	Follow-up time (month)	Outcome
Breck (5) 1956	A 21-year-old female	R	N^a^	Astragalectomy	24	A little, but not much pain; slight calcaneus deformity; no recurrence
Hull (13) 1977	A 14-year-old male	R	Talus and subsequently involving the calcaneus, tibiotalar joint, and the adjacent soft tissue	Curettage	21	Recurrence in the talus and calcaneus at 9 months. A talectomy, a wedge resection of the posterior calcaneus, and excision of involved soft tissue were performed
Moore (14) 1977	A 14-year-old male	L	N^a^	Curettage and allogeneic frozen-bone graft	36	No symptom; no recurrence
Ochsner (15) 1988	A 15-year-old male	R	2 cm	Curettage and cancellous bone graft from the distal tibia	27	No symptom; a full range of movement in his ankle and subtalar joint; no recurrence
Yu (16) 1996	An 18-year-old male	L	2.8 cm in maximal diameter	Curettage, autogenous bone grafting, and subtalar joint arthrodesis	27	The patient has resumed all activities without any difficulty; no recurrence
Sterling (17) 2002	A 20-year-old male	R	N^a^	Curettage and cancellous iliac crest bone graft	24	Some lateral ankle pain after heavy prolonged mechanical work; 15° dorsiflexion and 40° plantarflexion at both ankle joints; 75% normal eversion/inversion at the subtalar joint on the right compared with the left; no recurrence
Anderson (18) 2003	A 23-year-old male	R	2.5*2.0 cm, with the erosion of articular cartilage	Curettage, high-speed burring, and osteochondral autograft transfer from the lateral femoral condyle	24	No pain or swelling; a full range of motion of his ankle; no recurrence
Hassenpflug (19) 2007	A 18-year-old male	L	N^a^	Curettage and vascularized bone graft from the iliac crest	54	The patient was satisfied with the outcome; The range of motion of his left ankle was reduced to 5° dorsal extension and 20° plantar flexion; no recurrence
Zhang (20) 2012	A 22-year-old male	L	3.2*2.2 cm	Curettage and allogenic cancellous bone chip grafting	24	A little, but not much pain; no symptoms of instability; good mobility; no recurrence
Ningegowda (21) 2013	A 13-year-old male	L	N^a^	Curettage and fibular bone grafting	18	A full range of motion at the ankle, subtalar, and midtarsal joints; no recurrence
Sun (22) 2015	A 21-year-old male	L	3.0*2.0 cm	Curettage, high-speed burring, and bone cement implantation	6	No pain; The patient was satisfied with the functional outcome; no recurrence
Bahamonde (23) 2017	A 21-year-old male	R	Extending outside of bone boundaries and with a huge soft tissue mass and invasion of the adjacent calcaneus	En bloc talectomy, curettage, bone cement implantation, and tibiocalcaneal arthrodesis	132	The AOFAS hindfoot score was 83, with limitations regarding only hindfoot motion; no recurrence
Ryu (24) 2018	A 18-year-old female	L	2.5 * 4.1 * 2.9 cm	Curettage, burring, and fill as much of the body centrally with the cement and supporting the cortices with the autogenous cancellous bone graft from the ipsilateral iliac crest	12	No pain; the AOFAS hindfoot score was 100; no recurrence
Wagener (25) 2019	A 15-year-old male	L	4.0*3.0 cm	Debridement and microvascular iliac crest bone graft	60	The patient was very satisfied with the result and is playing soccer again; no recurrence
	A 20-year-old male	R	2.5*3.4*2.3 cm	Debridement and vascularized graft from the medial femoral condyle	72	The patient reported having no pain and no limitation in daily life activities, was highly satisfied; no recurrence
	A 33-year-old male	R	2.0*1.5*1.0 cm	Debridement and vascularized graft from the medial femoral condyle	36	Residual pain resolved after the screw was removed; no recurrence

Only references that reported clear outcomes of patients with talar chondroblastoma.

^a^ N, not mentioned.

Shears et al. (26) claimed that it is unnecessary to do a bone graft after benign talar tumor excision. However, in this small number of case series, we note that only two out of eight patients had a tumor volume larger than 10 cm^3^. Thus, this triggered concern about whether such findings also hold for those with relatively large-size lesions. In addition, it took 6–12 months for us to fully consolidate the defect, which delayed the recommendation of rehabilitation exercises to our patient. Moreover, early weight-bearing walking without the reconstruction of bony defects might cause talar collapse or fracture. Therefore, caution must be exercised because our patient had a large-sized talar chondroblastoma with a bony defect of 12.1 cm^3^. If a talar collapse or fracture happens, management would become much more difficult. Among multiple bone graft materials, autograft bone has the advantages of osteoconductivity, osteoinductivity, and potential osteogenicity (27–29). Therefore, it remains the gold standard for bone defect reconstruction. Similarly, autologous bone transplantation is the most widely used procedure in bone defect repair for talar chondroblastoma (10, 15–18, 21). Furthermore, a study reported that vascularized bone autograft was used to construct larger defects of the talar caused by bone tumor (19, 25). Although all these procedures are effective, potential pain and complications at the donor sites are worrying (30). Under such conditions, an allograft bone graft may be a good alternative (14, 20). Moreover, artificial bone graft substitutes such as the demineralized bone matrix or platelet-derived growth factor augmented ceramic granules showed good results compared with autologous grafts (31). In addition, bone cement implantation has been reported (22, 23). However, the major disadvantages of this procedure are the occurrence of thermal injury, secondary fracture, and osteoarthritis (32). It is of note that joint degeneration tends to be the case when the tumor is in close proximity to the articular cartilage (33). Van et al. (34) found that if the tumor–cartilage distance was ≤3 mm, the use of bone cement was more likely to result in joint degeneration. Thus, we did not use bone cement in our patient, as the remainder of the subchondral bone was extremely thin. We chose an allograft bone graft after considering the patient's apprehensions about extra donor site surgery and the resultant morbidities. On this basis, we combined allograft bone with PRP-FG to enhance tissue regeneration and repair (35). PRP-FG offsets the drawbacks of PRP, which lacks fixation and has poor tissue adhesive ability (36, 37). The final follow-up results of our case demonstrate the effectiveness of this procedure. However, a more prolonged observational follow-up is required to assess long-term results.

The technical difficulty of treating a talar chondroblastoma is that it lies in a deep and complicated position and is occluded by the tibial mortise and soft tissue of the posterior ankle. Open surgery, sometimes requiring osteotomy for better exposure of the lesion, may cause more trauma and induces subsequent tissue adhesions. In our case, posterior ankle arthroscopy offered a direct visualization of the surgical field, allowing for subsequent refined operation. At 2 weeks after arthroscopy, our patient was given the freedom of early partial weight-bearing and early mobilization. The patient was allowed to carry out the full weight-bearing activities of daily living at an earlier stage than that of open surgery. Our experience demonstrated the technical feasibility and superiority of arthroscopy. Arthroscopy is a safe, powerful, and minimally invasive technique offering good postoperative results. Studies have shown that arthroscopy has produced inspiring outcomes in benign bone tumors (38–42). More importantly, full functional recovery after a period of 8–12 weeks after arthroscopy has been observed. The application of arthroscopic treatment in chondroblastoma is listed in Table 2. In our case, we are pleased to report that arthroscopic treatment in talar chondroblastoma yielded good initial results. However, there is insufficient evidence of whether arthroscopic treatment of chondroblastoma won't decrease the tumor recurrence rate. Therefore, orthopedic surgeons must remain vigilant. A larger number of studies and a higher level of evidence are warranted to support this potential indication.

**Table 2:** Application of arthroscopic treatment in chondroblastoma previously reported in the literature.

Ref	Patient	Location	Follow-up time (month)	Recurrence	Complication
Cohen (43) 1992	1	Proximal tibia	12	No	No
Thompson (44) 1995	1	Femoral head	6	No	No
Stricker (45) 1995	3	Femoral head	4–38	No	No
Bal (46) 1995	1	Medial femoral condyle	60	No	No
Otsuka (47) 2002	1	Calcaneus	24	No	No
Prado (48) 2010	1	Dorsal medial cortex of the talar neck	3	No	No
Zoccali (49) 2012	1	Tibial spine area of the proximal tibia	12	No	No
Miyazaki (50) 2013	1	Medial femoral condyle	12	No	No
Errani (51) 2014	1	Lateral femoral condyle	12	No	No
Choi (42) 2014	1	N^a^	19	No	No
Farouk (41) 2018	3	Distal femur	26–45	No	No
Kellish (52) 2021	3	Distal femur	7–43	No	No
Acharya (53) 2022	1	Distal femur	84	No	No

^a^ N, not accessible.

## Conclusion

Posterior ankle arthroscopy with allograft bone graft and PRP-FG is a secure, motivating, and promising technique to treat chondroblastoma in the posterior talus, offering clinicians an excellent alternative to open surgery.

## Data Availability

The original contributions presented in the study are included in the article/Supplementary Material; further inquiries can be directed to the corresponding authors.
